# Portrait of Professor Wood

**Published:** 1860-05

**Authors:** 


PORTRAIT OF PROFESSOR WOOD.—Immediately before the Commencement of the University of Pennsylvania, March 15th, 1860, the graduates of the school presented, through one of their members, J. Campbell Shorb, M.D., of Maryland, the portrait of Professor Wood to the Board of Trustees, with a request that it should be placed in the Wistar and Horner Museum. The addresses delivered on the occasion have been published in pamphlet form, and will be read with interest by the profession. We extract from that of Dr. Shorb the subjoined passage:—

"The picture now stands before you. Of its artistic merits, of its truthfulness in delineating those loved and familiar features, we shall, of course, say nothing. It suffices that it meets with the approbation of its great original, and with this are we content. Our gratitude has not offered this tribute in the fear that time or oblivion would erase from the recollection of the medical world the name or mighty services of Dr. Wood. His name—his genius—is immortalized in his works, monuments "more lasting than brass," more durable than the lights and shades on yonder canvas. We have offered it, because it is most richly deserved, by every claim that moral worth, intellectual splendor, and highest professorial distinction, that unremitting zeal for our happiness and success, can have upon our veneration and gratitude."

The reply on behalf of the Board was made by Dr. La Roche, and abounds in many flattering but well-deserved allusions, expressed in chaste and glowing language, to the retiring Professor.

We give the remarks of Professor Wood in full:—

"When the heart is full, it is not always easy to command either our thoughts or the expression of them; and I am at a loss for words to say how much I am affected by your estimation of my character and services. I do not, however, wish it to be supposed that I consider myself entitled to the commendation which has been bestowed upon me. I am well aware how much allowance must be made, in reference to the sentiments of the class so glowingly expressed by their eloquent representative, for the warm and generous feelings of youth, and in reference to the approving remarks of the gentleman who spoke last, for the natural exaggerations of a friendship so long and so intimate as that which has existed between him and myself. Yet, I will not deny that I have done much hard work in the course of my life, and that, in doing it, I have endeavored to be guided by a sense of duty, and to aim at objects higher than those of a purely selfish character. Perhaps I may be allowed to draw from my experience a lesson, which may not be without its value to the younger among my hearers. One of the highest gratifications of my whole life, whether in its progress, or in a retrospect of the past, has been that derived from labor in the accomplishment of what I conceived to be useful ends. Let me, therefore, impress on you the importance to your own happiness, even in this world, of engaging energetically in useful labor, such as may be in accordance with your convictions of duty. Twenty years hence, should you follow this advice, you will, I confidently assure you, rejoice in having preferred a life of hard work directed toward noble objects, to one of idleness or self-indulgence.

"But I cannot leave this subject, after listening to the flattering compliments lavished on me, without confessing to you that I am conscious of many and great deficiencies; that I have let many opportunities of being useful escape, of which I ought to have availed myself; that I have left undone many things which I ought to have done, and, I fear, have done not a few things which would have been better left undone; that, in fine, in my course of life there has been much occasion for forgiveness from that Being, from whom alone forgiveness can come.

"To turn to the portrait which is the occasion of the present meeting, I cannot but feel very highly gratified by knowing that it is to be placed by the side of the portraits of those great men who founded and have supported this school, that, whenever you or your successors may walk through the splendid museum in which it is to be deposited, my remembrance will be called up in the mind in association with that of my illustrious predecessors. But, my friends, in a comparatively short period of time, the colors of these pictures will fade; in a few centuries the canvas itself will probably fall into dust, and perhaps the very names of these eminent persons may exist no longer in the memory of man. One recollection, however, will remain. The impression made upon my heart by your kindness will continue, I assure you, unimpaired throughout what may be spared to me of life; and, when life is passed, I sincerely hope that I may be permitted to bear it with me into eternity.

"Allow me once more most heartily to thank you, and to offer my sincere wish that you may enjoy long and prosperous lives, that all your just hopes may be realized, and all your noble aspirations gratified. Farewell."

Dr. Wood, it is understood, sent his resignation formally to the Board during the latter part of April. Early in the summer he contemplates leaving Philadelphia for Europe, to be absent about two years. We need hardly add that we most cordially wish him health and happiness during his foreign travels, and a safe return to his home.

